# A meta-analysis of the intervention effect of mindfulness training on athletes’ performance

**DOI:** 10.3389/fpsyg.2024.1375608

**Published:** 2024-05-31

**Authors:** Xing Wei Si, Zhen Kun Yang, Xia Feng

**Affiliations:** Henan Normal University, Xinxiang, Henan, China

**Keywords:** an athlete, mindfulness training, meta-analysis, sports performance, fluency

## Abstract

**Objective:**

To explore the intervention effect of mindfulness training on athletes’ performance using meta-analysis method.

**Methods:**

A total of 11 articles and 23 effect sizes were included through retrieval of Chinese and English databases, with a total sample size of 582.

**Result:**

Mindfulness training improves the level of mindfulness [SMD =1.08, 95%CI (0.30, 1.86), *p* < 0.01], fluency (The optimal competitive psychological state of the athlete, the athlete’s attention is all focused on the task, and other things no longer attract their attention) [SMD =1.47, 95%CI (0.87, 2.08), *p* < 0.001] and performance [SMD =0.92, 95% CI (0.40, 1.43), *p* < 0.01], reduced psychological anxiety [SMD = -0.87, 95% CI (−1.54, −0.20), *p* < 0.05], and all reached the level of large effect size.

**Conclusion:**

The effect of mindfulness training on athletes’ sports performance is effective, and it can be used as an effective psychological skill intervention method to improve athletes’ sports performance. In the future, we should further expand the sample size, strengthen the comparative study of different sports and intervention modes, and pay attention to the difference between the time effect and trait mindfulness level in fluency state.

## 1 Introduction

Athletic performance is the focus of attention in the field of competitive sports and competitions, and good athletic performance is the key to obtain excellent competition results. Athletes are highly stressed groups, and how to overcome adversity to obtain the best sports performance is one of the most difficult tasks in their sports career (Birrer and Morgan, 2010). Theoretical models related to sports performance, such as inverted U-shaped hypothesis, personal optimal functional area theory, drive theory, processing efficiency theory, attention control theory, etc., focus on the relationship between anxiety and sports performance under competition pressure from a specific aspect (Sun and Li, 2013, 2021). The integrated model of sports performance more comprehensively and systematically explains the different stages of sports performance and their influencing factors. The theoretical model holds that sports performance is determined by four factors, including physical fitness, environmental stimulation and requirements, personality and behavioral self-regulation, and sports performance is divided into preparation stage, performance stage and reaction stage after sports. In the preparation stage, athletes mainly face the internal and external demands that affect their competitive behavior. The cognitive, emotional and behavioral responses of individuals in the performance stage are easily affected by external events; The post-exercise reaction phase involves reactions to performance-related external consequences and internal processes (Gardner and Moore, 2007). As a result, sports performance refers not only to the athletic level behavior on the field, but also to the mental state and performance before, during and after the competition. The negative aspects of mental performance include anxiety, depression and Choking phenomenon, and the positive aspects include positive emotion, self-esteem, mindfulness level and fluency experience. Therefore, based on the integrated model of sports performance and the test indicators of quantitative intervention research, this study conducted a more comprehensive investigation of athletes’ sports performance from two aspects: psychological performance (psychological anxiety, level of mindfulness, fluency experience) and behavioral performance (performance).

In order to improve the athletic performance of athletes, the traditional control-based psychological skills training has been gradually applied to the field of sports, and has achieved certain results in reducing the “negative internal experience” and increasing the “positive state,” but its significant effect on the athletic performance of athletes has not been effectively supported, and it is faced with many difficulties (Noetel et al., 2019). First of all, the theoretical foundation is not solid. The “optimal state” theory holds that sports performance is dominated by potential psychological traits (such as anxiety, trait, self-confidence) or mental states (such as mood state, arousal state, fluency state), and the change of mental characteristics and mental states will also lead to the change of sports performance. The influence of environmental and learning factors on sports performance is ignored (Si, 2006; Moore, 2009). Secondly, the empirical effect is not ideal. Some researchers have used traditional psychological skills training to improve the performance of athletes, but there is no consistent empirical conclusion. Gardner and Moore conducted a qualitative study on the improvement of competitive sports performance by traditional mental skill training, and the results were not well proved. Finally, the practical operation is not strong (Gardner and Moore, 2006). What is the standard of optimal state for different individuals? How to operate to get the best state of mind? This is difficult to define in the actual application environment. In view of this, in recent years, in order to make up for the possible shortcomings of traditional mental skills training, mindfulness-based mental training has attracted more and more attention from sport psychology practitioners (Song and Zhang, 2020).

Derived from the Eastern Zen thought, mindfulness is closely related to religion, but it does not have the mysterious color of religion and has the characteristics of science. It is relatively popular in the West, mainly applied in clinical medicine, psychological intervention and other fields (Brensilver, 2011). Kabat-Zinn defines it as “mindfulness is the process of purposefully focusing attention on the present moment and becoming aware without judgment of the experience presented one moment after another” (Kabat-Zinn, 2003). With the rise of the third wave of cognitive behavioral therapy, mindfulness training has been widely used in the field of sports competition. From the perspective of theoretical models, attention control theory, interference theory and mind wandering theory all emphasize that athletes’ attention is susceptible to irrelevant information interference and lack of attention to the current movement process, which will cause anxiety and thus affect sports performance (Duan and Zhang, 2017). The mindfulness theory does not emphasize the control of the internal state and the acquisition of the best state, but focuses on the attention of their own situation and internal state without judgment and evaluation, so that athletes can focus on the current sports tasks, so as to effectively improve the psychological state of athletes and enhance their behavior. The main representatives are the mindfulness-accept- engagement training proposed by Moore and Gardner (2001) and Gardener and Moore (2004), the mindfulness exercise performance enhancement training proposed by Kaufman et al. (2009), the exercise mindfulness meditation training established by Baltzell and Akhtar (2014), and the mindfulness - acceptance - awareness - engagement training proposed by Si et al. (2014). A growing number of studies refer to the use of emerging technologies in mindfulness training. AI is expected to play a key role in future mental health programs. Artificial intelligence, through machine learning algorithms, can provide personalized recommendations, record progress, and provide real-time feedback to users. By identifying cognitive and behavioral patterns, AI applications can adapt based on real-time data from users. AI can identify the user’s personal preferences and interests and provide appropriate triggers to increase the user’s motivation to continue working toward the training goals (Mitsea et al., 2023). Mindfulness training based on virtual reality technology usually uses virtual reality technology to design and develop mindfulness training software, which is conducive to designing personalized courses, intelligent monitoring, emotion tracking and interesting games for patients’ individual conditions, so that patients have a high acceptance of mindfulness training based on virtual reality technology, which can improve patients’ negative emotions and fatigue (Wenjuan et al., 2024). From the perspective of empirical research, mindfulness training plays a positive role in helping athletes to accept their negative emotions (John et al., 2011), reduce anxiety (Watson, 2008; Zhao and Zhang, 2013) and improve experience acceptance (Gardner and Moore, 2006). In the field of competitive sports, mindfulness training intervention can effectively improve athletes’ mindful attention and awareness ability (Bernier et al., 2009), fluency experience, and athletic performance level (Wolanin, 2005). Mindfulness meditation is also able to improve attentional resource allocation (van den Hurk et al., 2010a), enhance working memory and executive ability (van den Hurk et al.,2010b; Jha et al., 2010), improve attention levels and motor skills (Cascante-Rusenhack et al., 2016). Foreign studies have shown that mindfulness is negatively correlated with job burnout, emotional/physical exhaustion, and will decline. Mindfulness training can alleviate burnout and chocking phenomenon (Vealey et al., 2014), improve athletes’ emotional state, enhance sports awareness and performance ability (Walker, 2013; Baltzell et al., 2014), and increase the frequency and duration of fluency (Aherne et al., 2011). Relevant domestic evidence also shows that mindfulness training can reduce pre-competition anxiety (Feng and Si, 2015), improve emotional state (Yang and Zhang, 2014), improve mindfulness level and sports performance (Zhong et al., 2013).

Overall, in recent years, the field of competitive sports has widely begun to apply mindfulness training to improve sports performance, and has achieved certain results. However, due to the short period of entry into this field, relative empirical studies are less than clinical intervention fields, and athletes belong to a special group with limited sample access, so the sample size of a single study is not large. In view of this, this study used meta-analysis method to obtain a larger sample and integrate relevant research results to systematically evaluate the intervention effect of mindfulness training on athletes’ sports performance, with a view to providing a basis for reasonable and effective psychological intervention methods for athletes.

## 2 Research methods

### 2.1 Literature screening

**Figure 1:**
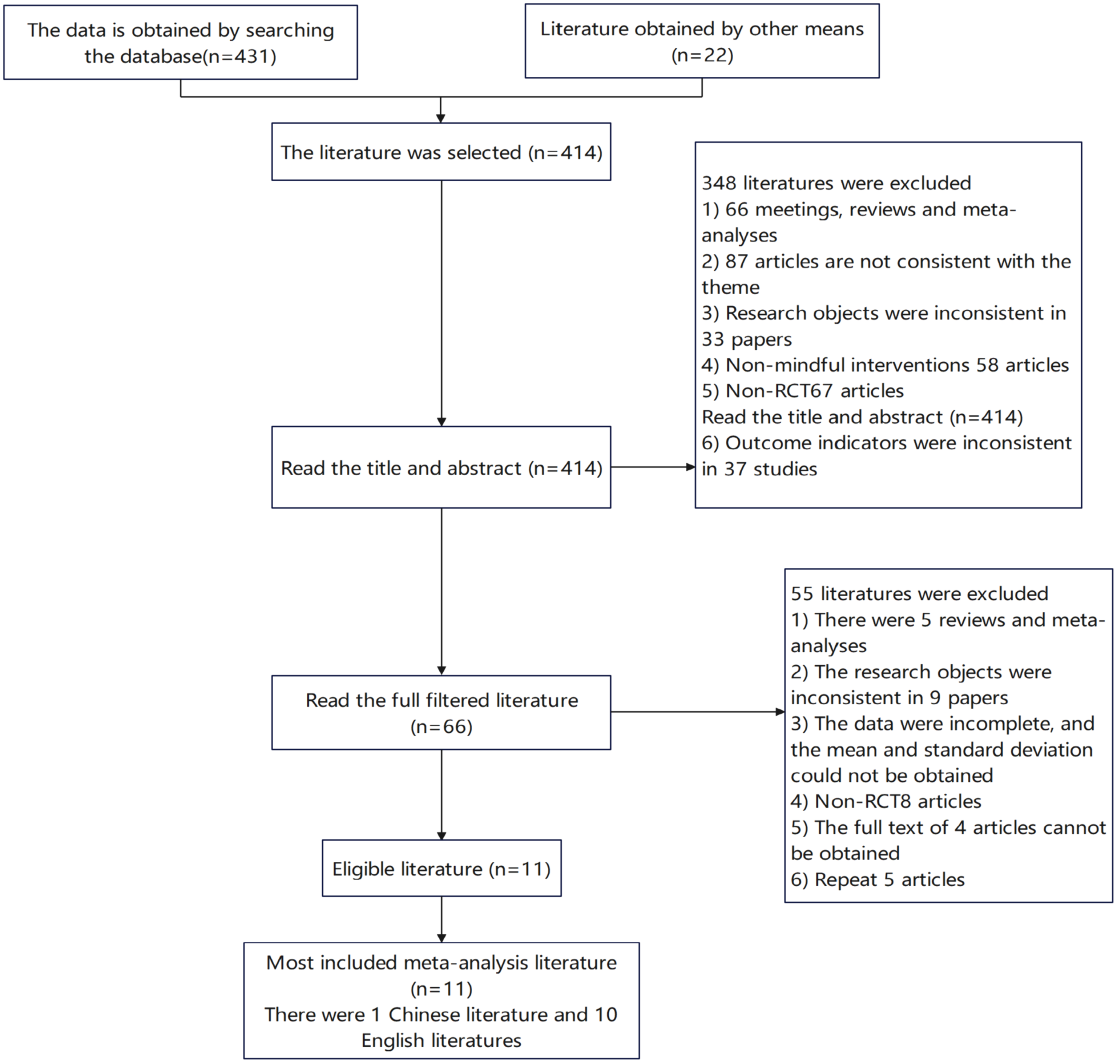
Flow chart of inclusion of literature screening.

This meta-analysis was conducted according to PRISMA guidelines (Page et al., 2021), and the searching protocol and inclusion criteria (detailed below) were decided by research team in regular meetings. Two researchers from the team conducted the literature screening. The key words for screening of English literature were selected from prior studies, and the variants of the terms were obtained from academic dictionary (e.g., Collins English Thesaurus). The terms of mindfulness, training, meditation, athletic performance, randomized controlled trail (RCT) and their variants were searched using Boolean formula in Chinese (i.e., CNKI, Wanfang, and VIP) and English sources (i.e., Web of Science, EBSCO, Science Direct, and Pubmed), please see Supplementary Table 1 for details of searching strategies. The literature screening phase ended on June 10, 2020. The title and abstract literature screening returned 181 Chinese literatures (limited core journals, CSSCI), 250 English literatures and 22 other sources were obtained Details of the procedure was presented in Figure 1.

### 2.2 Inclusion and exclusion criteria

#### 2.2.1 Inclusion criteria

The inclusion criteria were strictly in accordance with PICOS criteria (Amir-Behghadami and Janati, 2020). (1) The subjects were athletes, not limited to sports; (2) The interventions are mindfulness training, meditation or mindfulness-based methods; (3) The experimental design part of the included literature was randomized controlled experiment (RCT); (4) The outcome indicators were mindfulness level, happiness flow, competition pressure or anxiety, sports performance and achievement; (5) Sample size, average and standard deviation of experimental group and control group can be obtained.

#### 2.2.2 Exclusion criteria

The main exclusion criteria are: (1) experimental design non-randomized controlled experiment; (2) Non-athletes; (3) Non-mindful intervention; (4) Conference abstracts, dissertations, review articles, meta-analyses, duplicate publications, inability to obtain full text, etc.; (5) Inconsistent with the research theme; (6) Outcome indicators are inconsistent; (7) The data is incomplete, and the data needed for research cannot be obtained.

### 2.3 Literature screening and data extraction

The literature initially retrieved was imported into the literature management software, the duplicate literature was eliminated, and then the title and abstract were read to exclude irrelevant literature, and the full text was further read to determine the final literature included in the study. In the whole process, the two researchers independently screened and extracted the literature. In case of any disagreement, the included literature and its related information were finally determined through discussion or with the assistance of a third party (Figure 1). The extraction content mainly includes the first author, publication years, research methods, research objects, gender, age, sample size, intervention measures of the experimental group and control group, outcome indicators, etc.

Literature retrieval in the database was as follows: CNKI (*n* = 31), VIP (*n* = 5), WanFang (*n* = 145), Web of Science (*n* = 107), PubMed (*n* = 60), Science Direct (*n* = 74), EBSCO (n = 9).

### 2.4 Quality evaluation

**Figure 2:**
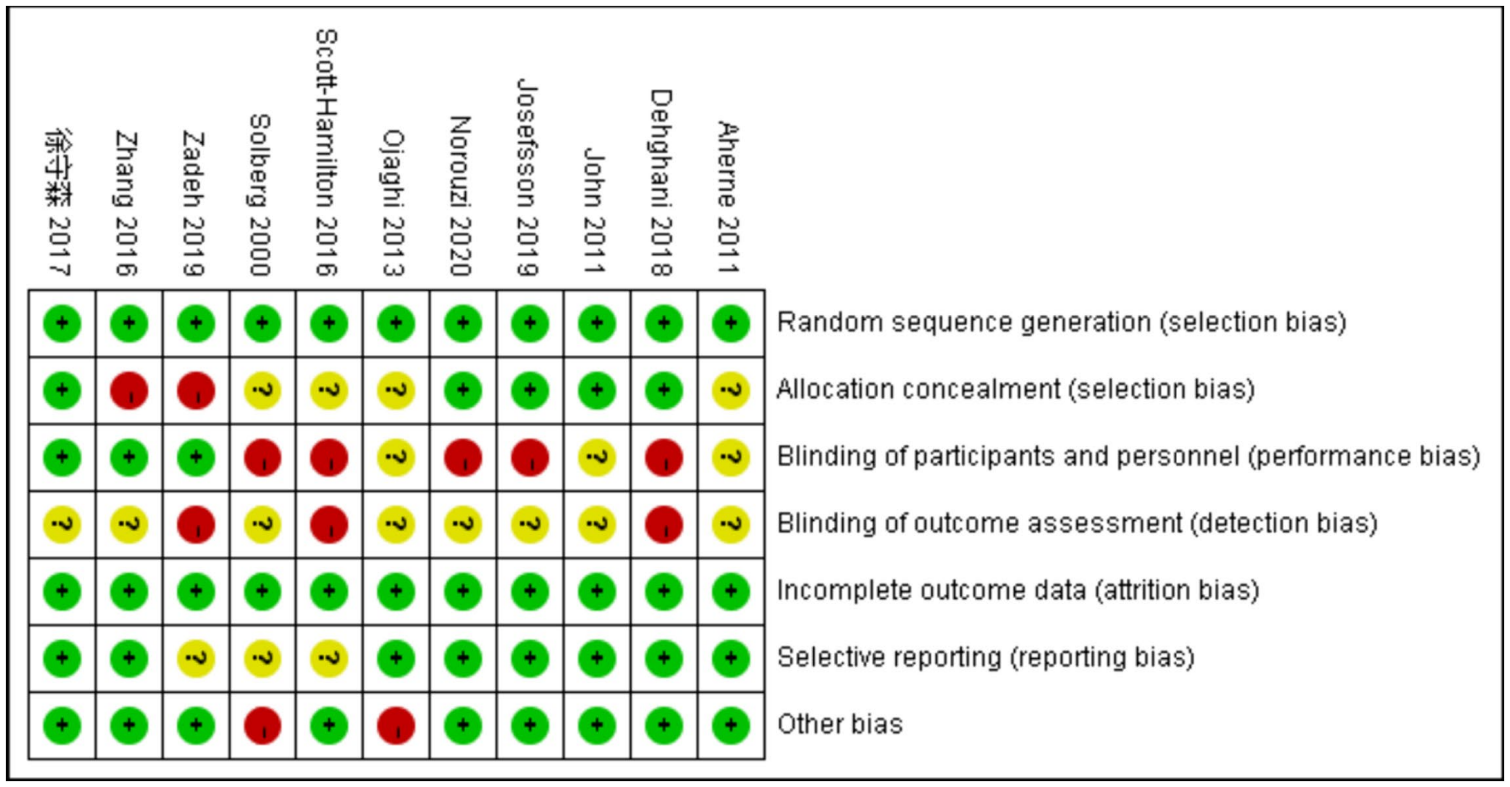
Risk assessment of the included literature.

The quality of the included literature was evaluated according to the evaluation criteria of the Cochrane Handbook 5.1.0. The evaluation includes the following aspects: (1) random sequence generation; (2) Distribution hiding; (3) Implement blind method for the implementers and participants; (4) Implement blind method for results evaluators; (5) Integrity of data results; (6) Selective reporting of research results; (7) Other sources of bias. A detailed description of those criteria can be found elsewhere (Higgins and Altman, 2008). The evaluation was completed by two researchers, and if there was any disagreement, the third researcher would discuss and decide together. The results are shown in Figure 2.

The three options of risk assessment were coded as followings: high risk (red; 0), unclear (yellow; 1), low risk (green; 2); the total score is ranged between 0 and 27, with higher score indicated higher study quality. The quantification of this assessment was used to examine whether the study quality contributes to the variance of the main outcomes. The meta regression was conducted for synthesis with more than five observations (i.e., mindfulness, sport anxiety, and performance), and permutation test was conducted to avoid the risk of overfitting. The results indicated that the quality of the study does not affect the main outcomes.

### 2.5 Outcome variables

The concept of “flow” refers to a highly desired but elusive state of mind characterized by total focus on the task at hand as well as enhanced skill performance. Elite athletes with high levels of personality mindfulness tend to experience flow. Some flow experts believe that flow can be difficult to achieve when a person is anxious because anxiety triggers negative self-conscious focus that disrupts concentration (Csikszentmihalyi, 1990; Jackson and Csikszentmihalyi, 1999), some flow literature suggests that the cognitive component of anxiety, rather than the physiological component, may be the most important cause of anxiety’s negative impact on flow (Jackson and Wrigley, 2004). Mindfulness may be a catalyst for flow, while mindfulness can also reduce the likelihood of anxiety, and a review of the mindfulness and sports literature has shown that increased mindfulness may lead to altered relationships with internal experiences such as anxiety (Gardner and Moore, 2012).

### 2.6 Data analysis

R (version 4.3.1) software was used to analyze outcome indicators of the included literatures. For continuous variables, Weighted Mean Difference (WMD) was used if the same measurement tools were used. If the measurement tools used are different, the Standard Mean Difference (SMD) is used. There are differences in the measurement tools used in the study, so the standardized mean difference (SMD) is used as the effect size indicator. Heterogeneity was tested by *I*^2^ statistic. When *p* ≥ 0.1 and *I*^2^ ≤ 50%, fixed-effect model was used for analysis. When *p* < 0.1 and I^2^ > 50%, the heterogeneity between the studies was indicated, and the random effects model was used for analysis.

## 3 Research results

### 3.1 Basic characteristics of the included research literature

A total of 453 literatures were preliminarily retrieved. Through the formulation of inclusion criteria and exclusion criteria, the search results were screened and read, and a total of 11 eligible randomized controlled trials were included (Table 1). Three studies included only men, one study included only women, and the rest included both men and women. All participants were athletes with an average age range of 16 to 67 years. The sample size was 582 people, including 305 men and 116 women. There were two literatures that did not report the number of gender column. The main intervention methods were mindfulness-accept-input training, mindfulness-stress reduction therapy, mindfulness-meditation training and other mindfulness-related intervention methods, while the control group mostly adopted no intervention, psychological skills training and educational lectures. Outcome indicators: level of mindfulness, fluency, psychological anxiety (degree) and academic performance. According to the definition of effect size by Cohen et al. effect size (0.2 ≤ ES < 0.4) is a small effect, effect size (0.4 ≤ ES < 0.6) is a medium effect, and effect size (ES ≥ 0.6) is a large effect, and *p* < 0.05 (Cohen, 1962; Wu et al., 2013).

**Table 1:** Basic features of the literatures included in this study.

Author and year of publication	Research method	Research object	N		Sex		Age (M ± SD)	Intervention measure		Outcome index
			E	C	male	female		E	C	
Zadeh et al. (2019)	Randomized control	Football player	22	22	44	0	E:23.77 ± 1.95C:24.86 ± 4.68	MAC 45 min/time 6 sessions/week 7 weeks	NT	①Mindfulness (MSPQ) ②Team performance
Norouzi et al. (2020)	Randomized control	Football player	20	20	40	0	34.05 ± 1.72	MBSR 90 min/time 16 sessions/week 8 weeks	NT	Anxiety (BAI)
Scott-Hamilton et al. (2016)	Randomized control	Cyclist	27	20	42	5	E:38.96 ± 12.40C:40.65 ± 10.88	MiCBT 65 min/time 7session/week 8 weeks	NT	① Mindfulness (FFMQ) ② Flow (DFS-2) ③ Anxiety (SAS-2)
Ojaghi et al. (2013)	Randomized control	Table tennis player	20	20	Failure to report		Failure to report	Mindfulness	NT	① Mindfulness (MAAS) ② Anxiety (CSAI) ③ Sport performance
Aherne et al. (2011)	Randomized control	Elite athlete	6	7	9	4	21.00 ± 1.68	Mindfulness and CD 60 min/time 2 days/week 6 weeks	NT	① Mindfulness (CAMS-R) ② Flow (FSS-2)
Solberg et al. (2000)	Randomized control	runner	11	10	Failure to report		39 ± 7	Acem Meditation 2.5 h/session 1session/week 7 weeks	ATPS	Anxiety (STAI)
Josefsson et al. (2019)	Randomized control	Elite athlete	36	32	25	33	E:20.9 ± 4.24C:21.0 ± 4.16	MAC 50 min/session 1session/week 7 weeks	PST	① Mindfulness (AMQ) ② Anxiety (DERS) ③ Sport performance
John et al. (2011)	Randomized control	shooter	48	48	96	0	29.5 ± 4.3	MMT 20 min/day 6 days/week 4 weeks	NT	Shooting performance
Zhang et al. (2016)	Randomized control	College darts thrower	22	21	16	27	19.23 ± 1.27	MAC 80-90 min/session 1session/week 8 weeks	SPL	① Mindfulness (FFMQ) ② Flow (SDFS) ③ Dart throwing performance
Dehghani et al. (2018)	Randomized control	College women’s basketball player	14	15	0	29	E:23.44 ± 0.49C:23.34 ± 0.34	MAC 1.5 h/session 8sessions	NT	Anxiety (SCAT)
Xu et al. (2017)	Randomized control	Collegiate golfer	33	18	33	18	E:21.55 ± 1.42C:21.78 ± 0.88	MAC 70 min/week 1session/week 6 weeks	NT	① Mindfulness (FFMQ) ② Anxiety (CSAI) ③ Sport performance

(1) E, Experimental group; C, Control group; M, Mean value; SD, Standard deviation. (2) MAC, Mindfulness-Acceptance-Commitment Approach; MBSR, Mindfulness-based Stress Reduction; MMT, Mindfulness Meditation Therapy; NT, No Treatment; ATPS, Autogenic Training Problem Solving; PST, Psychological Skill Training; SPL, Sport Psych Lectures; MiCBT, Mindfulness-integrated Cognitive Behavior Therapy program. (3) MAAS, Mindful Attention and Awareness Scale; FFMQ, Five Facet Mindfulness Questionnaire; MSPQ, Mindful Sport Performance Questionnaire; CAMS-R, The Cognitive and Affective Mindfulness Scale-Revised; AMQ, Athletic Mindfulness Questionnaire; DFS-2, Dispositional Flow Scale-2; FSS-2, Flow State Scale-2; SDFS, Short Dispositional Flow Scale; BAI, Beck Anxiety Inventory; SAS-2, Sports Anxiety Scale-2; CSAI, Competitive State Anxiety Inventory; STAI, State–Trait-Anxiety-Inventory; SCAT, Sport Competition Anxiety Test; DERS, Difficulties in Emotion Regulation Scale.

### 3.2 Publication bias test

**Figure 3:**
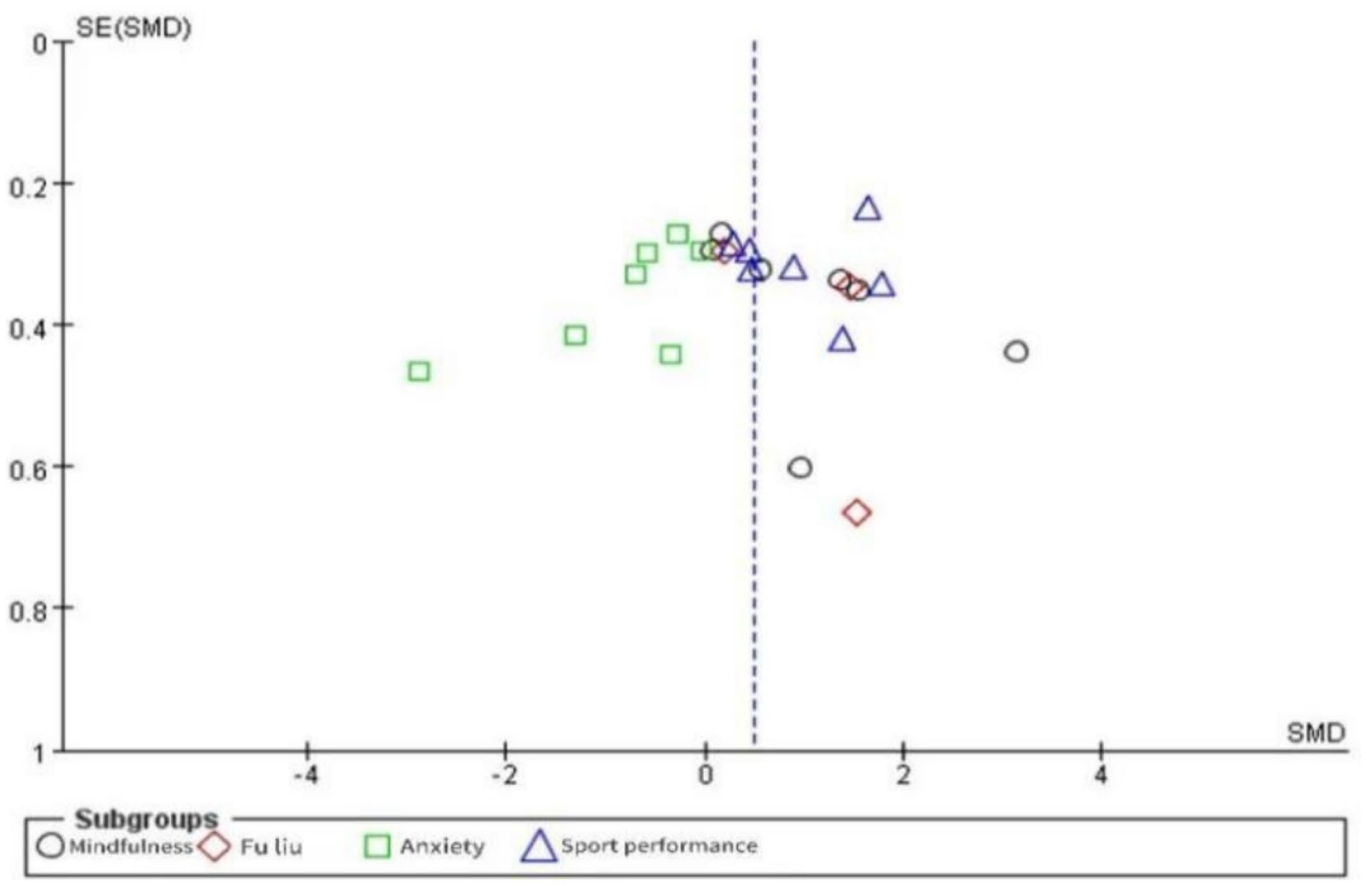
Shows a biased funnel diagram.

Generally, in meta-analysis, only when the number of studies is greater than 10 can a funnel plot be made to observe the publication bias of an article (Liu, 2011). A total of 11 literatures and 24 studies were included in this study, and publication bias test could be conducted if the conditions were met (Figure 3). As can be seen from Figure 3, the funnel plot is basically symmetrical, indicating that there is no significant publication bias in the study.

### 3.3 Sensitivity analysis

Sensitivity analysis was used to check the stability and reliability of meta-analysis or systematic review results, so as to provide guarantee for effective research. This study mainly adopted the method of gradually excluding literatures and changing the analysis model. The sensitivity analysis was carried out on 11 literatures and 24 studies included in the study, and the effect size was recalculated after each article was excluded to observe the heterogeneity. It was found that after the Scott-Hamilton study was excluded, and statistical heterogeneity decreased to a non-significant level, *I*^2^ = 0% (I^2^ < = 50%), chi-square test *p* = 0.93 (*p* > 0.1), see section 3.2 for details. There was no significant change in other results, indicating that the results of the meta-analysis in this study were credible.

## 4 Results of meta-analysis

### 4.1 Meta-analysis of mindfulness training on athletes’ mindfulness level

**Figure 4:**
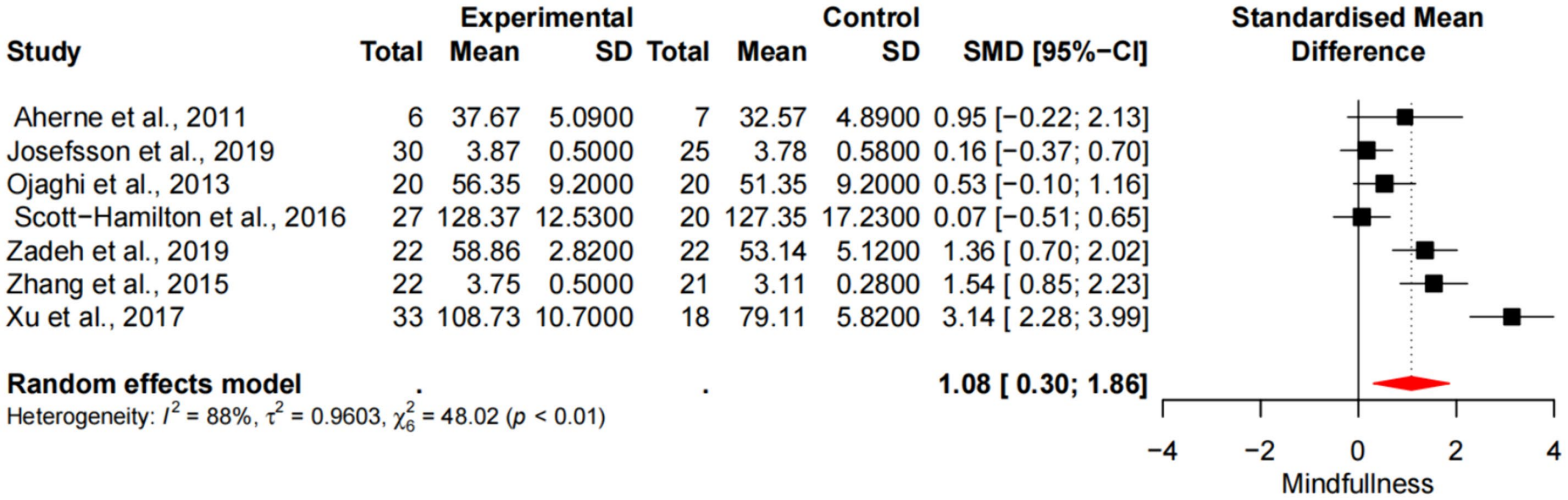
Forest diagram of the effect of mindfulness training on the level of mindfulness of athletes.

Seven studies demonstrated the impact of mindfulness training on the level of mindfulness in athletes, including a total sample size of 293 people. There was heterogeneity in the included studies (*p* < 0.01, *I*^2^ = 88%), which was analyzed by random effects model. The results showed (Figure 4) that there was a statistically significant difference in the level of mindfulness between the experimental group and the control group [SMD =1.08, 95%CI (0.30, 1.86), *p* < 0.01], and the average effect size was 1.08, p < 0.01, reaching the level of large effect size.

### 4.2 Meta-analysis of mindfulness training on athletes’ fluency status

**Figure 5:**
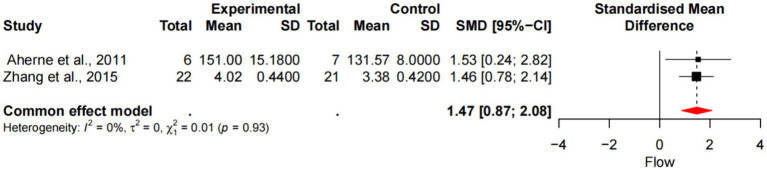
Forest diagram of the effect of mindfulness training on athletes’ fluency.

Two studies demonstrated the effect of mindfulness training on the fluency of athletes, with a total sample size of 56 people. No heterogeneity was identified after the removal of the study by Scott-Hamilton (*p* = 0.93, I^2^ = 0%), which was analyzed by fixed effects model. The results showed (Figure 5) that the experimental group and the control group had a significant difference margin in fluency [SMD =1.47, 95%CI (0.87, 2.08), *p* < 0.001], and the average effect size was 0.99, *p* = 0.05, reaching the level of large effect size.

### 4.3 Meta-analysis of mindfulness training on psychological anxiety of athletes

**Figure 6:**
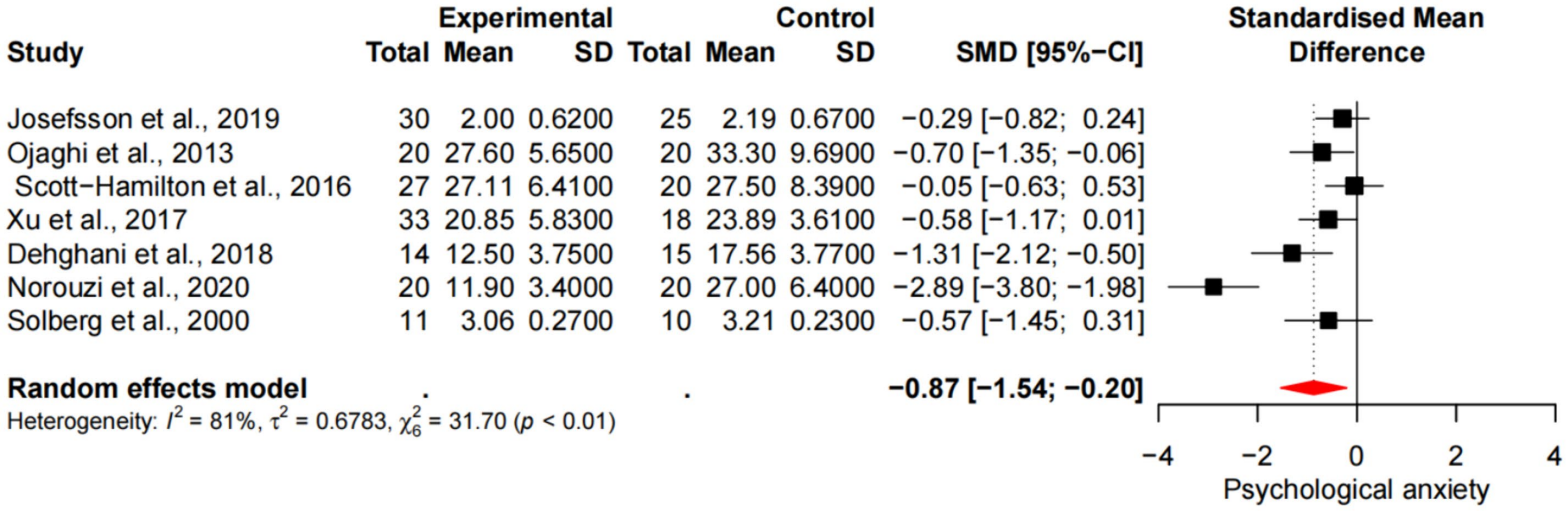
Forest diagram of the effect of mindfulness training on psychological anxiety of athletes.

Seven studies demonstrated the effects of mindfulness training on psychological anxiety in athletes, including a total sample size of 283 people. There was heterogeneity in the included studies (*p* < 0.1, *I*^2^ = 81%), which was analyzed by random effects model. The results showed (Figure 6) that there was a statistically significant difference between the experimental group and the control group in psychological anxiety (degree) [SMD = -0.87, 95%CI (−1.54, −0.20), *p* < 0.05], and the average effect size was 0.83, *p* < 0.01, reaching the level of large effect size. “-” indicates that the psychological anxiety of the individual has been relieved after the experiment.

### 4.4 Meta-analysis of mindfulness training on athletes’ performance

**Figure 7:**
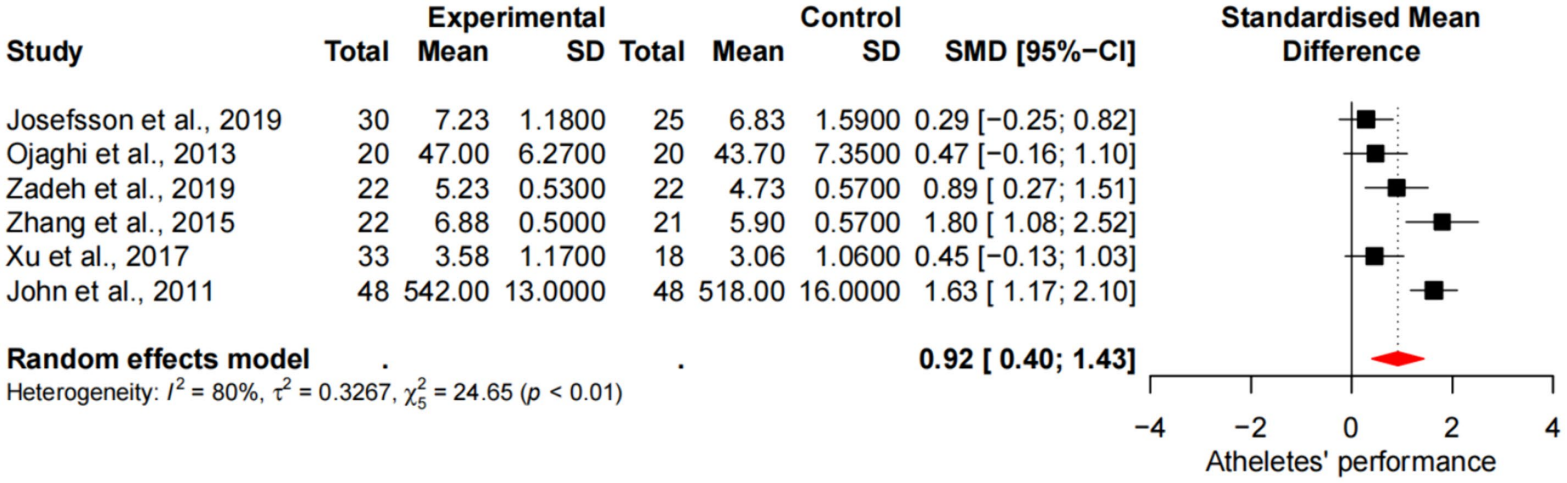
Forest diagram of the effect of mindfulness training on athletes’ performance.

Six studies demonstrated the impact of mindfulness training on athlete performance, including a total sample size of 329 people. There was heterogeneity in the included studies (*p* < 0.1, I2 = 80%), which was analyzed by random effects model. The results showed (Figure 7) that the difference in performance between the experimental group and the control group was statistically significant [SMD =0.92, 95%CI (0.40, 1.43), *p* < 0.01], and the average effect size was 0.92, *p* < 0.001, reaching the level of large effect size.

## 5 Discussion

### 5.1 Impact of mindfulness training on the level of mindfulness of athletes

Mindfulness intervention has been widely used in medical and health care systems and has made a positive contribution to promoting health levels (Kiely, 2016). As more and more research confirms the beneficial effects of mindfulness, the use of mindfulness training methods in competitive sports and sports has been surging in recent years. Mindfulness is an open, accepting, non-judgmental state of awareness in which individuals focus their attention on the present moment, on internal and external things and experiences. The ease with which the individual maintains this state of awareness is a manifestation of good control of attention. Mindfulness is a psychological tactic whose main purpose is to increase an individual’s attention to the present moment through a variety of methods (Davis and Hayes, 2011). Individuals with high levels of mindfulness are able to separate their attention from the meditative state and focus highly on the task at hand (Gardner and Moore, 2007). Attention control, mental adjustment and mental toughness are the three key areas for athletes. At present, most athletes realize the necessity of physical and technical training, but the recognition of the benefits of mental training is not yet in place (Ferraro and Rush, 2000). Bishop et al. believe that mindfulness consists of two parts: one is to focus attention on the current task; the other is to adapt to the situation in the present moment according to the individual’s experience ability, and the whole process is full of curiosity, openness and acceptance (Bishop et al., 2004). Holzel pointed out that mindfulness meditation can enhance attention control, emotional regulation, and self-awareness (Hölzel et al., 2011a). Studies on the mechanism of attention control and self-awareness have shown that both short-term and long-term mindfulness training can cause changes in the structure of the brain’s white matter and gray matter. It can be seen that mindfulness training can affect the brain and nervous system and promote the learning and mastery of motor skills (Hölzel et al., 2011b). Chinese scholar Yang Shu et al. pointed out in a study on the Impact of mindfulness cognitive Intervention on psychological indicators related to stress coping in high-level athletes that mindfulness cognitive intervention training for athletes can effectively improve their mindfulness level (Yang and Zhang, 2014). Li Jieling et al. conducted a sustained attention intervention study on badminton team athletes and college students, and found that mindfulness training can effectively cope with mental wandering, thus improving athletes’ sports performance (Li et al., 2017). Duan Zaifu et al. also came to the same conclusion in his article “A New Perspective for Choking in Competition: the Wandering Theory of Mind” (Duan and Zhang, 2017). This study integrates 7 data related to the impact of mindfulness training on the level of mindfulness of athletes, and also confirms that mindfulness training is an effective way to improve the level of mindfulness of athletes [SMD =1.08, 95%CI (0.35, 1.82), *p* = 0.004], and can improve the attention of athletes when applied to sports. Promote athletes to achieve good performance in the arena.

### 5.2 Influence of mindfulness training on athletes’ fluency

The fluid experience proposed by Csikszentmihalyi has contributed greatly to positive psychology and is now an important branch of the field (Csikszentmihalyi, 1990). In the process of skill display, athletes will have a peak moment of high concentration of attention, focus on the current moment, physical and mental integration, self-confidence doubling, mental relaxation, and performance beyond normal. This state is usually called the “best zone,” and the state in this region is the state of fluency (Young and Pain, 1999). The so-called state of fluency is the state of consciousness in which the athlete is fully engaged in an athletic task and performs at the best athletic level (Jackson and Marsh, 1996). Fluid state is an optimal psychological state, which is closely related to sports performance and performance. Therefore, it is very important to promote the appearance of athletes’ optimal psychological state in the field of competitive sports. In college physical education, smooth experience has been proved to play a role in the field of sports for many times (Jackson and Roberts, 1992; Zhang and Ma, 2007). Participants experience the state of flow, which is conducive to increasing their interest and enthusiasm in sports activities and improving their participation behavior (Mandigo and Thompson, 1998). As for the research on mindfulness training and fluency experience, (Hasker, 2010) conducted mental skill training of mindfulness training and imagery training on athletes of different disciplines, and found that athletes in the mindfulness group obtained a higher sense of peak experience, and the group’s subjective assessment performance improved significantly. Bernier et al. (2009) pointed out that focused athletes are more likely to experience smooth experience and feel the fun and satisfaction brought by sports. Schwanhausser (2009) suggests that the increase in propensity and fluency is related to the mindfulness-acceptance intervention style. Foreign studies have also found that fluency experience is negatively correlated with anxiety, perfectionism and thought disruption, and positively correlated with confidence and mindfulness (Russell, 2001), and the significant correlation between the two has positive significance for the use of mindfulness psychological intervention (Kee and Wang, 2008). Based on MAIC, Chinese scholar Bu Danran tested the impact of mindfulness intervention on tennis players’ mindfulness level, fluency experience and sports performance. According to the data, athletes’ mindfulness level, mood state and fluency experience of traits have been significantly improved (Bu, 2015). For the relationship between the two, Yin Yuanmei intervenes in shooting athletes with the help of mindfulness training program, introduces emotional regulation self-efficacy, and confirms its regulatory role between mindfulness and smooth experience (Yin, 2015). In summary, using different mindfulness training methods to intervene athletes in different sports, the results all show that mindfulness training can improve athletes’ fluency (Li and Sun, 2000; Hasker, 2010; Liu et al., 2016). In this study, meta-analysis method was used to integrate relevant data for quantitative analysis, and the results showed that mindfulness training was conducive to improving athletes’ fluency state and experience [SMD =0.99, 95%CI (0.02, 1.96), *p* = 0.05]. Both of them showed marginal significance (*p* = 0.05), which may be related to the fluency state being a higher psychological state of competitive sports psychology, and it may also be related to the level of athletes’ trait mindfulness and the time effect of mindfulness training. It is suggested to increase the relevant empirical research in this field.

### 5.3 Influence of mindfulness training on psychological anxiety of athletes

The arena is like a battlefield, and athletes inevitably have pressure in this special environment, resulting in anxiety (Anshel and Wells, 2000). Competition pressure has a negative impact on athletes’ competitive performance on the arena. Therefore, it is particularly important to find out the source of pressure and relieve pressure and anxiety. The source of pressure mainly comes from internal and external factors, including coaches, referees, players, opponents, spectators, time, environment, competition results, etc. The main internal factors are the athlete’s ability level, concentration, self-awareness, self-confidence, sports recovery and so on. Some of these factors are uncontrollable, in the face of uncontrollable factors, the most important thing is to learn and have the ability to self-regulate the physiological and psychological state, give full play to their own level, and achieve the best sports performance (Scoffier et al., 2010). Daily mental training is a compulsory course for every athlete, and having a good mental state is the premise of excellent performance in the competition. As a psychological intervention method, mindfulness training makes up for the shortcomings of traditional psychological skills training. It does not emphasize the establishment of the best psychological state, does not control and change the internal negative state, and advocates the attention to the present and acceptance without judgment. The mindfulness re-perception model holds that mindfulness contains three elements: attention, purpose and attitude, and its core mechanism is re-perception (Shapiro et al., 2006). Later, Brown et al. concluded that through re-perception or de-self-centralization, mindfulness training can strengthen non-judgmental evaluation of experience, weaken emotional bias toward stimulus perception, and help individuals avoid ineffective and counterproductive adverse emotional states as much as possible (Brown et al., 2007). Relevant foreign studies have shown that mindfulness meditation training can enhance positive emotions, and with the increase of trait mindfulness, positive emotions are gradually enhanced (Nyklíček and Kuijpers, 2008; Zautra et al., 2008; Orzech et al., 2009). Relevant meta-analysis found that mindfulness training, as a clinical intervention, can help patients alleviate a variety of mental health problems and improve mental function (Baer, 2003). Thompson et al. confirmed that mindfulness training can improve athletes’ mental state during sports, increase their acceptance of experience, improve happiness and mobility, and reduce the risk of anxiety, stress and burnout (Thompson et al., 2011). Studies have found that MAC training can alleviate and reduce state anxiety during competition (Piet et al., 2012; Forsyth and Eifert, 2016). Domestic research has also found that mindfulness training can reduce the level of trait anxiety and self-awareness of athletes, and improve the tendency to deal with problems and emotions (Zhao and Zeng, 2013). With the enhancement of mindfulness tendency during sports, the brain will focus on the current sports task, ignore the factors unrelated to the game, temporarily forget the pain, anxiety, pressure and other factors, and devote themselves to it, so as to obtain the best sports performance (Brotto et al., 2012). It can be seen that mindfulness has a regulating effect on athletes’ emotions (Chen et al., 2011). This study sorted out the data of the included literature, and 7 studies discussed the impact of mindfulness training on the psychological anxiety of athletes. Through the integration of the data, it was found that mindfulness training could reduce the psychological anxiety of athletes, and the result was statistically significant (*p* = 0.006), which was consistent with previous studies by scholars. Therefore, mindfulness training can be applied to the field of sports as an alternative psychological intervention to traditional mental skill training.

### 5.4 Impact of mindfulness training on athletes’ performance

Competitive sports are full of competition and challenges, athletes are under various pressures and events at the same time, but also constantly improve their motor skills, in order to obtain good sports performance and excellent sports results. Sports performance is most intuitively reflected in the athletic performance of the athletes, therefore, to improve the athletic skill level of the athletes and promote their athletic performance is the main goal of the coach and the team. Mindfulness training in the field of sports began with the article “Mindfulness Meditation Training in University and Olympic Rowing” by Western scholars such as Kabat Zinn. The emergence of mindfulness training methods has gradually become an alternative to mental skill training in the traditional sense of improving sports performance (Moore, 2003). The application of mindfulness training in sports environment can improve athletes’ performance, mental state and overall happiness (Gardner and Moore, 2012). At present, domestic and foreign studies have proved the positive role of mindfulness in improving attention, regulating emotional status, and improving sports performance (Sappington and Longshore, 2015; Huang and Ning, 2017) conducted a systematic review of relevant studies on the intervention of mindfulness training on athletes, and concluded that mindfulness-based mental training methods can help improve athletes’ competitive performance. Jouper and Gustafsson (2013) uses mindfulness training with shooters to help them regain their confidence and be fully engaged (John et al., 2011). Timothy conducted a questionnaire survey on the rowers’ self-efficacy, team efficacy belief, mindfulness and fluency. The research showed that self-efficacy and team efficacy belief were significantly correlated with mindfulness, fluency and sports self-confidence, and the higher the level of mindfulness, the higher the level of self-efficacy and team efficacy belief. It is more helpful for athletes to improve their performance (Pineau et al., 2014). Wan Chunmei conducted an 11-week intervention on the young athletes of the national diving team with mindfulness training, and the results showed that after the intervention, the level of mindfulness of the athletes was improved, and the test scores were also improved, indicating that mindfulness training is conducive to improving sports performance (Wan, 2019). This study conducted data integration of the included studies. Six studies discussed the intervention effect of mindfulness training on athletes’ sports performance. The comparison between the mindfulness training experimental group and the control group also showed consistent research conclusions with other scholars, which proved that mindfulness training can improve athletes’ sports performance and performance [SMD =0.92, SMD =0.92, SMD =0.92, SMD =0.92, SMD =0.92, SMD =0.92, SMD =0.92, SMD =0.92, SMD =0.92, SMD =0.92, SMD =0.92, SMD =0.92, SMD =0.92. 95% CI (0.39, 1.45), *p* = 0.0006], the results were statistically significant. Therefore, the application of mindfulness-based psychological intervention to the field of competitive sports has achieved remarkable results and can be regarded as an effective way to improve performance.

### 5.5 The effect of different mindfulness training on training results

In order to make the course schedule fit with the time characteristics of sports training, different mindfulness training courses are also arranged differently. Table 2 summarizes and compares the course schedule design of these training methods:

**Table 2:** Course time design of main mindfulness training methods.

	MAC	MSPE	MMTS	MAIC
Time span	7 ~ 12 weeks	4 weeks	6 weeks	7 ~ 8 weeks
Class hour	7 ~ 12	4	12	7 ~ 8
Length per class hour	1 h	2.5 h	0.5 h	1 h

From the findings of the research results, the effects of the four training methods on the level of mindfulness and acceptance have been effectively proved, but the empirical research results related to the effects of different mindfulness training methods are also different. For example, it is found that both MAC training and MAIC training are effective in improving athletic performance. Long-term follow-up studies have also proved that MSPE training is effective in improving athletic performance (Thompson et al., 2011). However, MMTS training is not designed to directly target the impact on sports performance, so the results of relevant studies do not reflect its impact on sports performance.

Athletes who participate in mindfulness training must meet the criteria of not having any physical or mental illness, hearing impairment, and not smoking, taking drugs and drinking alcohol. There are also potential risks in the experiment. Participants did not consume caffeine or alcoholic beverages in the 12 h before the experiment, and did not exercise in the 12 h before the experiment, especially during the test period (John et al., 2011). In fact, multiple studies have shown that raising awareness too closely during mindfulness practices can make mental health conditions worse, including increasing the risk of depression, anxiety, schizophrenia, or substance abuse, and decreasing the ability to tolerate pain. Mindfulness researchers and project developers have recognized that while reversing the flaws in MRPs can improve well-being, little attention has been paid to the negative effects of overdoing these processes on well-being (Britton, 2019). Therefore, the duration and frequency of mindfulness training should be strictly grasped during the experiment to minimize the probability of risk.

## 6 Limitations and future studies

### 6.1 Limitations

This study integrates the research results of mindfulness training on athletes’ sports performance through meta-analysis, which is larger than the sample size of a single empirical study and the research results are more convincing. However, there are also shortcomings in this study: (1) Only published literatures were included in this study, and unpublished studies were not taken into account. The research data are not comprehensive enough, which will affect the reliability of the research results to some extent. (2) The athletes included in the study were engaged in different sports, and the intervention methods, intervention cycles, time and frequency were also different, which may be the reason for the high heterogeneity. (3) The protocol of this meta-analysis was not registered, which may affect the quality and replicability of the findings. However, this study applied stringent strategies to maximize the retrieval of relevant articles and to prevent the inclusion of duplicated studies. For example, articles authored by the same individuals, affiliated with the same institution, or sharing the same trial registration ID were considered at high risk of duplication. Additionally, full information regarding sources and corresponding search strategies were reported to facilitate perfect replication of this study, thus mitigating this limitation to some extent. (4) The number of studies included in this meta-analysis was limited, especially for the outcome of flow. The power to detect dose–response effects was similarly limited for this reason. Specifically, it remains unclear to what extent mindfulness could maximize its effects on sports performance. Only a few studies examined this feature(Wang et al., 2023)., which warrants further investigations to validate, the heterogeneity was high in most of the syntheses, which may affect the external validity of our findings.

### 6.2 Future studies

Based on the research prospects of this study: (1) It is suggested to further strengthen the practical application research of mindfulness training, expand the sample size, and strengthen the comparative study between different exercise items and intervention modes. (2) Studies on mindfulness training and sports performance mostly focus on athletes. Future studies may consider focusing on college students majoring in physical education to provide more powerful evidence for mindfulness training and sports performance. (3) It is suggested to strengthen the empirical research on fluency from the differences in trait mindfulness level and time effect of mindfulness training. (4) Future studies should try the relevant studies and compare the effects of different mindfulness training on the end goal and whether there are different effects on the training program. (5) The application of the new technology is still limited, and it is hoped that future research will apply VR to mindfulness training.

## 7 Conclusion

Meta-analysis evidence shows that mindfulness training can significantly improve athletes’ sports performance, which is embodied in the level of mindfulness, fluency, psychological anxiety and other psychological performance, as well as performance and other behavioral performance. As a psychological intervention method, mindfulness training can improve the level of mindfulness, fluency and performance of athletes, reduce the level of psychological anxiety, and the effect size reaches the level of large effect value. The course span is 7 to 12 weeks, the number of lessons is 7 to 12 times, each lesson length of 1 h mindfulness training is effective in improving athletic performance, and helps athletes to be in the best condition. It is worth noting that mindfulness training has a marginal effect on improving fluency.

## Data availability statement

The original contributions presented in the study are included in the article/Supplementary material, further inquiries can be directed to the corresponding author.

## Author contributions

XS: Conceptualization, Data curation, Formal analysis, Funding acquisition, Investigation, Methodology, Project administration, Resources, Software, Supervision, Validation, Visualization, Writing – original draft, Writing – review & editing. ZY: Conceptualization, Data curation, Formal analysis, Funding acquisition, Investigation, Methodology, Project administration, Resources, Software, Supervision, Validation, Visualization, Writing – original draft, Writing – review & editing. XF: Conceptualization, Data curation, Formal analysis, Funding acquisition, Investigation, Methodology, Resources, Supervision, Validation, Visualization, Writing – review & editing.
